# The Causation of Cancerous and Other Tumours

**Published:** 1901-03-23

**Authors:** 


### THE CAUSATION OF CANCEROUS AND OTHER TUMOURS.

The thoughtful and interesting address which was recently delivered by Professor Adami[1] before the Yale University Alumni Association on the causation of cancerous and other new growths is very suggestive as showing that, whether tumours are malignant or benign, they depend on an alteration in the direction in which their constituent cells dispose of their energy; an alteration which is common to all forms of tumour. But when we come to ask, What is the cause of this altered display of energy? when we inquire, Why do cells which have hitherto been lying idle, or have been performing special functions, take on a tendency to growth, and thus by repeated multiplication produce a tumour? we find no answer. It is something, however, to have it laid down with some degree of positiveness, that, even if some microbic influence should prove to be the starting point of the process, in certain cases this is only one among a series of forms of stimulus by which cells may be urged to take on growth, instead of continuing to carry on function, and that it is this habit of growth, by whatever hereditary tendency, or by whatever alteration of environment it is set up, which determines tumour formation. What is the cause of the cells taking on this habit is the question at issue, the question which has not so far been solved. It is clear enough that certain tumours are due to independent cell growth without microbic intervention. On the other hand there are tumours which assuredly are initiated by microbic intervention. Of both these facts there can be no doubt; but between these two extremes we have a very broad debatable territory wherein we encounter the main mass of neoplasms, and the question is, to which class do they belong? Another and perhaps a more important question is, Can we determine anything in common—a common denominator as it were—associating these various groups? Now the more one studies what occurs in the normal life of the various tissues, the more obvious is it that multiplication and the active performance of other function by the cell are incompatible. A cell may perform its fully differentiated function, or it may grow, but it will not do both. For a cell to divide actively there must be at least temporary arrest of the specific as distinct from the vegetative functions; while conversely arrest or disturbance of the specific functions of the cell, if of such a nature as not to arrest vegetative activity, favours cell multiplication. The nuclear activities unemployed in one direction become diverted into another. This arrest or disturbance of specific function is a matter of environment, and thus we see that if the cell be not called upon to perform its normal functions, be not called upon to work after previous stimulation has led to increased absorption and anabolism, that cell is in a favourable condition to divert its energies from function to proliferation.

Thus we come to a common starting point for all forms of tumour. This starting point is that condition in which the cells of the part, whether functional or lying latent, upon being stimulated, and as a consequence undertaking active assimilation and anabolism, cannot from their relations carry on their peculiar functions to a corresponding extent, so that the accumulated energy is instead utilised in mitotic changes and cell division rather than in the normal katabolism. When once started this tendency to proliferate, instead of to functionise, becomes a "habit of growth," and thus tumour formation, once started, tends to go on. Whatever the origin of the tumour proper, however it is started, what makes the tumour is the assumption by the primary cells of that tumour of the habit of growth in place of the habit of work; and according to the extent of this replacement, so do we get the various grades of tumour formation, from the most benign to the most malignant.

When, however, we come to ask again what is the cause of this change, from habit of function to habit of growth, we get no clear answer. It has something to do with the environment of the cell; maybe something to do with lessened call for its own special kind of work, as we may imagine to be the case with certain cells at or after the climacteric; maybe some interference with function in consequence of microbic invasion; maybe this, maybe that—we cannot tell. But, says Professor Adami, "the greatest benefit to the patient, the greatest triumph and satisfaction to the practitioner, will for yet some years to come be the recognition and successful removal of malignant tumours at the earliest possible date—aye, and, what is more, the removal of benign tumours in general before they have taken on possible malignant characters." Malignancy is not of necessity the microbic or other influence by which the cell activities were first turned aside from function or from latency to growth, but is the "habit of growth" so set up, and, like other bad habits, this should be nipped in the bud.

[1] Brit. Med. Jour., March 16.

